# Supplementary material for: The Role of Greenery in Stress Reduction among City Residents during the COVID-19 Pandemic
Source: Int J Environ Res Public Health. 2023 May 16;20(10):5832. doi: 10.3390/ijerph20105832 (PMC10218576; doi:10.3390/ijerph20105832)
Supplement: Supplementary file 1 [file ijerph-20-05832-s001.zip › ijerph-2303439-supplementary.pdf]

Table 1. Content and characteristics of geosurvey questions

The content of the question	Type of an answer to the question
"What is the level of stress related to the COVID-19 pandemic that you perceive/ fell?"	– closed question,<br>– a dichotomous slider on a scale from "0" to "10", where "0" meant "no stress" and "10" - "very high" level of stress
"How has your perceived level of stress changed due to the COVID-19 pandemic?"	– closed question,<br>– choice 1 out of 3 answers: "decreased", "no change", "increased".
„How stressful are you for you during the pandemic - please move the cursor on the axis:<br>– the presence of the virus;<br>– uncertainty regarding changes in the scope of restrictions (introduced overnight)<br>– concern about limiting contact with family/ friends<br>– concern of losing one's own job or that of another close relative<br>– concern about the loss of part of the income<br>– concern about price increases<br>– concern about the need to provide childcare in the event of the closure of nurseries/schools/kindergartens<br>– concern about being quarantined<br>– concern about change of working mode to remote<br>– concern about providing all household members with conditions for remote work/ learning (computer equipment, rooms)<br>– concern about using public transport<br>– closure of sports clubs, gyms, swimming pools and fitness clubs<br>– closure of gastronomical facilities<br>– closure of cultural and entertainment facilities (cinema, theatres, etc.)<br>– restrictions on leaving apartments and using public spaces<br>– difficulties in accessing health care (family doctor, specialist doctor, hospital treatment, etc.)<br>– the need/ obligation to wear protective masks<br>– concern about non-compliance with restrictions by other people<br>– difficulties and risks related to movement (domestic/ international trips)"	– closed question,<br>– evaluation of each of the proposals included in the list,<br>– a dichotomous slider on a scale from "0" to "10", where "0" meant "no stress" and "10" - "very high" level of stress

„Do you perceive/ feel any of the effects of excessive stress:<br>– weakness and general unwillingness to act;<br>– problems with concentration/ memory;<br>– anxiety attacks;<br>– sleep disorders;<br>– lack of appetite;<br>– compulsive eating;<br>– growing family conflicts;<br>– growing conflicts at work;<br>– growing neighbourly conflicts”	– closed question,<br>– evaluation of each of the proposals included in the list,<br>– answers to choose from: "yes" or "no".
"The use of which elements of the city structure increases the stress connected with the pandemic that you perceive/ feel, and which reduces it:<br>– public transport stops, stations<br>– small shops/ service points<br>– large shopping malls<br>– markets<br>– churches<br>– gyms, fitness clubs, swimming pools and other sports facilities<br>– outdoor recreation areas (outdoor gyms, sports fields, tennis courts, playgrounds, etc.)<br>– green areas (parks, recreational and leisure compact green areas, allotment gardens)<br>– cultural and entertainment facilities (cinema, theatres, clubs, discos)<br>– gastronomical facilities (cafes, restaurants, pubs)<br>– collective accommodation facilities (hotels, motels, guesthouses)<br>– public administration facilities (ZUS – Social Security Work, offices, municipal offices, town halls)<br>– health care facilities (clinics and doctor's offices, hospitals)<br>– public spaces - place, source (being among people)<br>– office spaces<br>– pandemic advertisements”	– closed question,<br>– evaluation of each of the proposals included in the list,<br>– answers to choose from: "reduces stress", "no change", "increases stress".
"Please indicate on the map from 1 to 5 places where you relax or reduce the level of perceived stress."	– indicating on the interactive map of the city from 1 to 5 specific locations (green pin),<br>– for each indicated location (pins), a specific type of place (e.g. park, restaurant, gym) had to be specified in the empty field displayed next to it (next to map and pins).

"Please indicate on the map from 1 to 5 places that stress you or increase the stress you perceive/ feel related to the pandemic."	– indicating on the interactive map of the city from 1 to 5 specific locations (red pin),<br>– for each indicated location (pins), a specific type of place (e.g. park, restaurant, gym) had to be specified in the empty field displayed next to it (next to map and pins).
„What helps you reduce the level of stress you perceive/ feel during the pandemic:<br>– vaccination against COVID-19<br>– following news/information about the pandemic in the media (television, press, etc.)<br>– undertaking/ continuing hobby activities (manual works, etc.)<br>– direct contact with relatives (family, friends)<br>– using social media,<br>– watching movies, series, programs, etc.<br>– staying in public spaces (among people)<br>– contact with a pet<br>– staying in a fitness club/gym<br>– yoga/meditation<br>– shopping in the mall/shopping centre<br>– outdoor physical activity (walking, running, cycling, etc.)<br>– staying among greenery (parks, gardens, green areas)<br>– looking at greenery/water/nature,<br>– working in the garden, caring for plants”	– closed question,<br>– evaluation of each of the proposals included in the list,<br>– answers to choose from: "yes" or "no".
” In what directions should the city change to make it more resident friendly (reducing their stress) in the event of future pandemics?:<br>– built-up areas vs. green areas (parks, boulevards),<br>– un arranged greenery (forests, meadows) vs. arranged greenery (parks, , recreational and leisure compact green areas, squares, boulevards)”	– closed question,<br>– - dichotomous slider on a 10-point scale.
